# Micro Finite Element models of the vertebral body: Validation of local displacement predictions

**DOI:** 10.1371/journal.pone.0180151

**Published:** 2017-07-11

**Authors:** Maria Cristiana Costa, Gianluca Tozzi, Luca Cristofolini, Valentina Danesi, Marco Viceconti, Enrico Dall’Ara

**Affiliations:** 1 Department of Oncology and Metabolism, University of Sheffield, Sheffield, United Kingdom; 2 INSIGNEO Institute for In Silico Medicine, University of Sheffield, Sheffield, United Kingdom; 3 Zeiss Global Centre, School of Engineering, University of Portsmouth, Portsmouth, United Kingdom; 4 School of Engineering and Architecture, Alma Mater Studiorum–Università di Bologna, Bologna, Italy; 5 Department of Mechanical Engineering, University of Sheffield, Sheffield, United Kingdom; University of Zaragoza, SPAIN

## Abstract

The estimation of local and structural mechanical properties of bones with micro Finite Element (microFE) models based on Micro Computed Tomography images depends on the quality bone geometry is captured, reconstructed and modelled. The aim of this study was to validate microFE models predictions of local displacements for vertebral bodies and to evaluate the effect of the elastic tissue modulus on model’s predictions of axial forces. Four porcine thoracic vertebrae were axially compressed *in situ*, in a step-wise fashion and scanned at approximately 39μm resolution in preloaded and loaded conditions. A global digital volume correlation (DVC) approach was used to compute the full-field displacements. Homogeneous, isotropic and linear elastic microFE models were generated with boundary conditions assigned from the interpolated displacement field measured from the DVC. Measured and predicted local displacements were compared for the cortical and trabecular compartments in the middle of the specimens. Models were run with two different tissue moduli defined from microindentation data (12.0GPa) and a back-calculation procedure (4.6GPa). The predicted sum of axial reaction forces was compared to the experimental values for each specimen. MicroFE models predicted more than 87% of the variation in the displacement measurements (R^2^ = 0.87–0.99). However, model predictions of axial forces were largely overestimated (80–369%) for a tissue modulus of 12.0GPa, whereas differences in the range 10–80% were found for a back-calculated tissue modulus. The specimen with the lowest density showed a large number of elements strained beyond yield and the highest predictive errors. This study shows that the simplest microFE models can accurately predict quantitatively the local displacements and qualitatively the strain distribution within the vertebral body, independently from the considered bone types.

## Introduction

Throughout life the structural stability of bones is compromised by a reduction in bone mineral density (BMD) due to the changes driven by ageing and diseases. Vertebral fractures are common and related to different pathologies such as osteoporosis and bone metastases [1,2]. The current clinical methods used to evaluate pathological risk of fracture are mainly based on areal measurements of BMD and qualitative assessments of radiological data which *per se* are not enough to provide an objective and accurate prediction of bone strength [3]. On the other hand, the relationship between bone morphology and mechanics has been driving the development of more accurate and reliable micro Finite Element (microFE) models to predict non-invasively the local and structural properties of bone under loading.

MicroFE models based on high-resolution imaging (i.e. High Resolution peripheral Quantitative Computed Tomography, HR-pQCT, and micro Computed Tomography, microCT) can resolve bone structural heterogeneities and are used to better understand bone deformation under complex loading. Such models are typically generated by segmentation of the images, and conversion of bone voxels into linear hexahedral elements [4–6]. Due to the long computation time required to run non-linear models with several millions of degrees of freedom (DOF), typically microFE models at the organ level are run within the elastic regime. Furthermore, the bone tissue is usually considered as isotropic and homogeneous [7–12], with the Poisson’s ratio equal to 0.3 and the Young’s modulus estimated from microindentation measurements [11,13], or through back-calculation procedures [4,9,14,15]. Specifically, the local elastic properties of vertebral bone reported in the literature showed a wide range of values: mean values (±standard deviations) from 5.7±1.6GPa ([16] from back-calculation procedures) to 12.3±1.0GPa ([13]from microindentation tests performed on wet bone structural units, BSU) (Table 1).

**Table 1:** Overview of the elastic modulus of human vertebral bone tissue reported in the literature from wet microindentation tests performed at the BSU level, or from back-calculation procedures in combination with microFE models.

Reference	Method	Sample Size	Bone Type	E_tissue_ [GPa] (range)	Dimensional level of μFE models	Imaging technique (voxel size)	μFE models (element size)
*Wolfram et al*. *(2010)* [17]	Wet microindentation ^**a**^	N = 104	Trab	12.0±1.0 (N/A) ^**b**^	N/A	N/A	N/A
*Wolfram et al*. *(2010)* [13] ^*c*^	Wet microindentation ^**a**^	N = 30	Trab	12.3±1.0 (N/A) ^**b**^	Biopsy	μCT (12μm)	Linear (36μm)
*Hou et al*. *(1998)* [16]	Back-calculation	N = 28	Trab	5.7±1.6 (2.7–9.1)	Biopsy	μCT (50μm)	Linear (50μm)
*Ladd et al*. *(1998)* [15]	Back-calculation	N = 5	Trab	6.6± 1.1 (5.4–7.7)	Biopsy	SR-μCT (23μm)	Linear (23μm)
*Pahr et al*. *(2011)* [9]	Back-calculation	N = 37	Trab/Cort	8.8±N/A (N/A)	Vertebral body	HR-pQCT (82μm)	Linear (82μm)

^a^ Penetration Depth equal to 2.5μm, loading rate = 120mN/min, holding time 30s

^b^ Values of elastic tissue modulus computed from indentations performed along the axial direction

^c^ In this study predictions of microFE models of trabecular bone set with an average tissue modulus measured from wet microindentation tests provided excellent quantitative predictions of structural stiffness measured in compression (concordance correlation coefficient of 0.97)

N/A Information not available.

MicroFE models predictions of structural properties depend on the defined tissue properties [14,18,19]. The specificity of the back-calculated tissue’s elastic modulus to the imaging procedure, anatomical site, and modelling approach [4,9], reduces its applicability and generalization. However, microFE models defined with an elastic tissue modulus based on the average value measured through wet microindentation tests have been shown to provide accurate estimations of apparent stiffness for trabecular bone biopsies scanned with 12μm voxel size and extracted from human vertebrae tested in compression (concordance correlation coefficient equal to 0.97) [13]. Nevertheless, from the literature it is not clear if this value can be used also for whole vertebral bodies. MicroFE models generated from HR-pQCT images with 82μm voxel size were found to predict up to 84% of the variability in bone stiffness and up to 92% in variability of bone strength when compared to *ex vivo* compression tests of human vertebral bodies [9,20]. However, a good quantitative agreement of structural stiffness (Slope = 0.88, Intercept = 0.07GPa) was obtained only once a back-calculated tissue modulus was used [9].

Digital Volume Correlation (DVC) can provide an accurate measurement of the 3D displacement field in bone tissue given two microCT images of the undeformed and deformed specimens [21,22], and has been used to validate displacement predictions of microFE models for trabecular bone specimens scanned with voxel size equal to 10μm and 35μm [7,23]. In particular, it has been demonstrated that in order to obtain proper correlations between the displacement values measured with DVC and predicted with microFE, the boundary conditions in the models need to be interpolated from the DVC displacement field in order to correct for potential experimental artifacts in the *in situ* time lapsed mechanical testing. The DVC approach has been also used to study the failure behavior of vertebral bodies [24–26] and trabecular bone tissues [27]. Jackman et al. used DVC to compare the predicted local axial displacements of QCT-based FE models of vertebral bodies tested up to failure, showing a wide range of predictive ability of the best models (Pearson correlation coefficients between 0.40 and 0.95, derived from the plots) and large median errors (45–50%, estimated from the plot) [28].

The accuracy of homogeneous microFE models in predicting bone mechanical properties is mostly affected by their ability of modeling bone geometry, microstructure and material properties [11,29]. Therefore, inaccuracies depend on the type of bone (i.e. differences in bone architecture and volume fraction) [15,29], the used imaging protocols [30], which should minimize discretization errors such as partial volume effect [31,32], and the assigned tissue modulus. To the authors’ knowledge there is no evidence in the literature about quantitative comparison of specimen-specific microFE models predictions of local displacements at the organ level, where the accuracy of microFE models relies also on the ability of the imaging procedure to capture both cortical and trabecular bone microarchitectures. Moreover, linear microFE models predictions of structural properties have been only validated for input images with 82μm voxel size, leaving unknown their predictive ability if based on images with higher resolution. In particular, considering the ability of this method to account for bone microarchitecture and its potential to analyze the effect of musculoskeletal pathologies and related interventions [33–35], it is very important to understand if the models can accurately predict the local displacements in the elastic regime and provide reasonable estimations of structural properties.

Therefore, the aim of this study was to evaluate the ability of specimen-specific microFE models to predict the local displacements across the whole vertebral body, and in particular on cortical and trabecular compartments, measured with *in situ* compressive tests and DVC analyses. Furthermore, in order to evaluate the effect of the tissue modulus on the structural properties of vertebral bodies, the axial forces predicted by the microFE models were compared to those experimentally measured.

## Materials and methods

**Fig 1:**
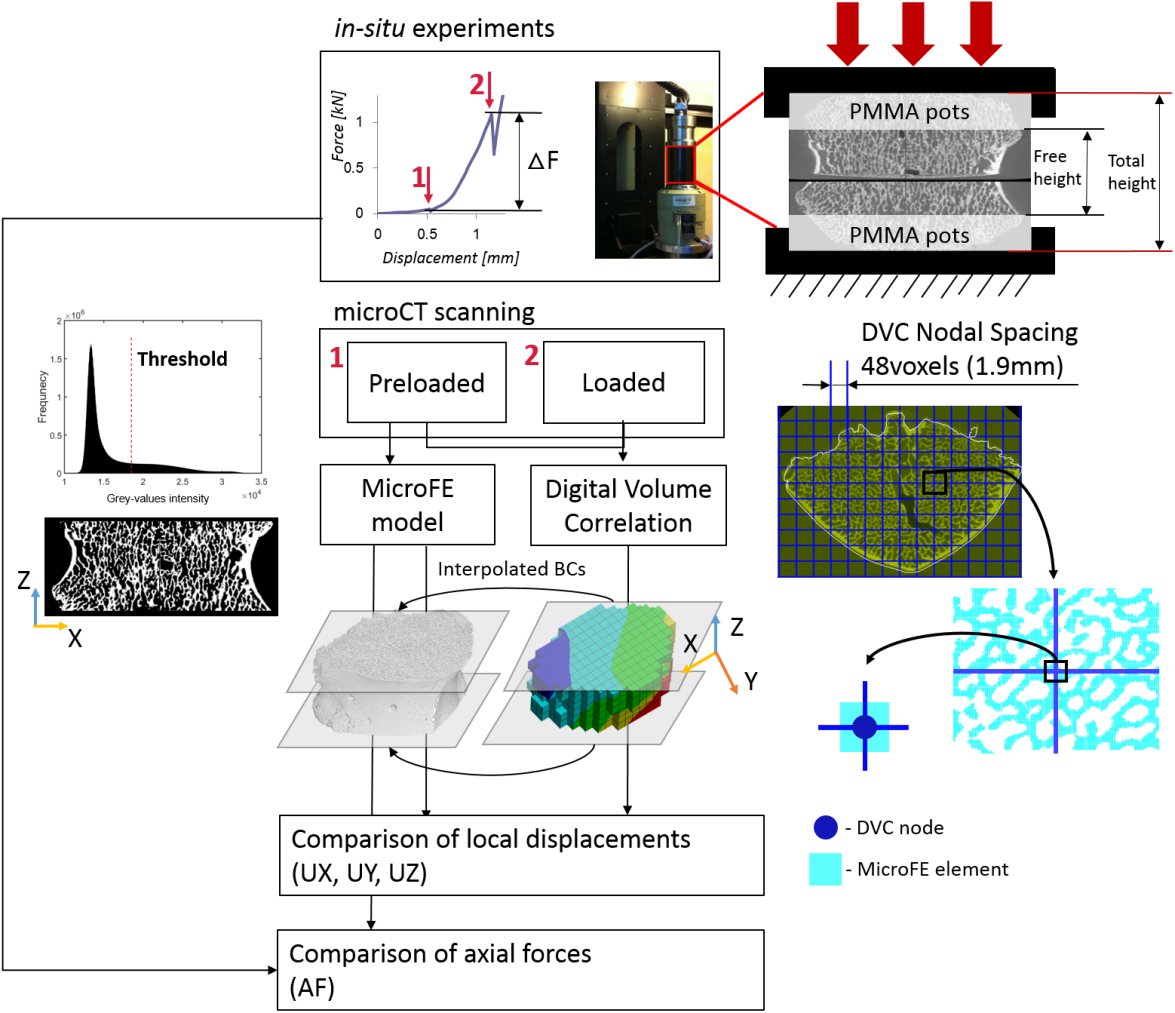
Workflow used to compare predicted and experimental local displacements and axial forces predicted. An example of the step-wise load displacement curve is reported on the top highlighting the Preloaded (1) and Loaded (5% apparent strain, 2) conditions. A picture of the loading jig and a scheme of the sample fixation are reported on the top-right corner. The Digital Volume Correlation (DVC) algorithm was applied to the Preloaded and Loaded images to calculate the map of displacement in the whole vertebral body. MicroFE models of the vertebral body between the PMMA pots were generated from the preloaded image after the application of a single level threshold chosen from the analyses of the frequency plot of the grey-values and visual inspection. The displacement values at the top and bottom layer of the microFE models were assigned by interpolation of the DVC measurements in those planes. Displacements along the axial (Z) and transverse (X, Y) directions were compared between microFE predictions and DVC measurements at the nodes of the DVC grid that lay within microFE elements. Predicted axial forces were compared to those measured from the experimental load-displacement curves (ΔF).

In order to validate the predictions of the microFE models for porcine vertebral bodies we used a similar workflow as presented by [7] (Fig 1). Briefly, *in situ* compressive tests were performed within a microCT system that was used to acquire the geometry and microstructure of preloaded and loaded specimens as described in [25]. A DVC algorithm was applied to preloaded and loaded images to obtain the displacement fields. MicroFE models were generated from the preloaded images and displacements were imposed according to the DVC output at the boundaries. The predicted local displacements were compared to those experimentally measured with DVC in the middle of the specimen. Predicted and measured axial forces corresponding to the deformed state were compared as well.

### Specimen’s preparation

Four thoracic porcine vertebrae (T1-T3) were harvested from animals (females, approximately 9 months old, approximately 100 kg in weight) that were destined to alimentary purposes. Endplates, adjacent growth plates and surrounding soft tissues were removed and approximately 20% of the most caudal and cranial remaining portions of vertebral bodies were embedded in poly-methyl-methacrylate (PMMA). The spinous processes were used as reference to center and align the specimens along the transverse plane using a protocol adapted from [36]. Afterwards, the posterior arches were also removed.

### Scanning and *in situ* mechanical testing

An *in situ* mechanical loading device (CT5000, Deben Ltd, UK; nominal precision of axial displacement and force measurements were 10μm and 50N, respectively) was used to axially compress the specimens inside the microCT scanner. The two flat parallel external surfaces of the embedding material were positioned between the loading plates of the jig. A sandpaper disk was applied between the embedding material and the bottom loading platen to avoid relative rotations of the loading device. The free height of each specimen (i.e. distance between the internal surfaces of the embedding material, see Fig 1) was measured with a caliper. The specimens were compressed in displacement control at a loading rate of 0.1mm/s while immersed in a physiological saline solution. The vertebral bodies were scanned with a microCT system (XTH225, Nikon Metrology, UK) in a preloaded condition (50N in compression, in order to avoid moving artifacts during the microCT scanning) and after a 5% nominal global strain was applied considering as initial height the free height of specimens (loaded condition, Fig 1). The scanning was started approximately 15min after each compression step in order to reduce the effect of relaxation. Each image was acquired with an isotropic voxel size of approximately 39μm, and reconstructed after applying a median filter (kernel 3x3) on the projections (CTPro, Nikon Metrology, UK). The scanning parameters were: voltage of 88kV, current of 110μA, exposure time of 2s, and rotational step of 0.23° over 360° total rotation. The scanning time was approximately 90min for each step. For more details about the experimental procedure please refer to [37].

### Properties of the specimens

The free height of each specimen was computed as the mean distance between the top and bottom embedded pots measured with a caliper in three different positions (lateral left, lateral right, anterior and posterior). The total height of each vertebra was determined from the reconstructed microCT images. The preloaded and loaded images were cropped in order to remove image artifacts on the top and bottom slices (3–12% of the total height of the images). From each cropped preloaded image a specimen-specific mask was created by defining an initial contour of the entire bone structure applying a low threshold value and by using dilation and filling morphological functions (MATLAB 8.5, MathWorks, Inc., USA). To avoid modelling the portion of the bone within the embedding material, which had attenuation similar to the surrounding saline solution, the middle 50% (in total height) portion of the preloaded step of each specimen was cropped together with the masks in order to compute the total bone volume fraction (Tot.BV/TV), dividing the volume of bone voxels (BV) by the total volume within the mask (TV). A single threshold value was chosen visually for each portion of the preloaded image by comparing cross-sections of binary and grey scale images. Then a connectivity filter was applied to remove the voxels without face connectivity [7] to obtain the binary images required for the computation of the morphometric parameters and for the generation of the microFE models. To estimate the morphology of the trabecular bone for each specimen, four regions (5x5x10mm^3^) centered with respect to the mid cross-sectional plane were cropped in the lateral left, lateral right, anterior and posterior locations. For each region trabecular bone volume fraction (Tb.BV/TV), thickness (Tb.Th), separation (Tb.Sp), and degree of anisotropy (DA) were computed using the BoneJ 1.4.1 plug-in [38] on ImageJ 1.50e software (Table 2).

**Table 2:** Properties of the specimens.

Specimen ID	Level	Free Height [mm]	Voxel size [μm]	Tot.BV/TV [%]	Tb.BV/TV^a^ [%]	Tb.Th ^a^ [μm]	Tb.Sp ^a^ [μm]	DA ^a^
**S#1**	T3	12.9	39.0	41.3	41.5±2.4	217±39	419±138	0.65±0.03
**S#2**	T2	12.6	38.6	40.3	41.4±1.6	241±42	465±136	0.67±0.04
**S#3**	T1	10.8	38.6	32.7	32.9±3.6	198±37	503±154	0.53±0.05
**S#4**	T3	13.3	38.6	48.6	48.4±4.6	239±53	396±122	0.65±0.10

^**a**^ measurements performed on four sub-volumes in the lateral left, lateral right, anterior and posterior locations of the vertebral body.

Data reported as mean ± standard deviation.

### Experimental displacement field computed by digital volume correlation

The elastic image registration toolkit ShIRT-FE (Sheffield Image Registration Toolkit, University of Sheffield, UK) was used to find the full-field displacements over the entire specimen during the mechanical testing. The registration was applied to the cropped preloaded and loaded images using only the information within the mask, in order to reduce the effect of image noise outside the border of the specimens. Details of the DVC algorithm can be found in [39]. Briefly, ShIRT overlaps to the 3D images a grid with nodes spaced by a selected “Nodal Spacing” (NS). Through the recognition of 3D features the software computes the nodal displacements mapped between the images at different deformation stages. The DVC grid is then converted into an 8-noded hexahedral mesh, the displacement field measured from DVC is imposed to the mesh as boundary conditions and is then imported to an FE software package (*ANSYS*® Academic Research, Release 15.0) to compute the strain field. A NS equal to 48voxels (approximately 1872μm) was chosen as the best compromise between precision and spatial resolution of the DVC approach (precision errors below 3.7μm for displacements [40] and approximately 100μɛ for strains [41]).

### Micro Finite Element modelling

Each microFE model was generated by converting every bone voxel within the middle 50% of the total height of each specimen (computed from the preloaded images, Fig 1) into an 8-nodes linear hexahedral element. MicroFE models and DVC displacement maps were referred to the same reference system. The boundary conditions (BCs) of the microFE models were assigned by trilinear interpolation of the DVC displacement field [7,23]. Homogeneous and isotropic material properties were assigned to every bone element considering a tissue elastic modulus (E_t_) of 12.0GPa [17] and a Poisson’s ration equal to 0.3. Moreover, a back-calculated tissue modulus equal to 4.6GPa was also determined as the best least square fit between predicted and experimental axial forces for the four specimens. The experimental axial force (ΔF) was computed as the difference between the peak force measured at the loaded step (i.e. 5% apparent strain) and the force measured at the end of the relaxation period of the preload step (see Fig 1). From the microFE models, the total axial force (AF) was computed as the sum of the axial forces obtained from the top surface nodes (i.e. closer to the fixed loading platen). Experimental and numerical results of local displacements were compared in all nodes of the DVC grid which lay within a microFE element (number of comparison points for the specimens were between 130 and 226). In order to reduce the effect of the boundary conditions the comparison was performed within the middle 70% (in height) of the microFE models. For all analyses the Z direction is representative of the axial axis of the vertebral body. X and Y refer to transverse directions without a precise anatomical reference. MicroFE models and DVC analyses were based on the original microCT images without applying any rotation, in order to avoid potential errors induced by image interpolation.

In order to investigate the results for trabecular and cortical sub-structures separately a mask of the cortical shell was generated (CTAnalyzer software version 1.16.4.1, SkyScan product provided by Bruker) for each specimen. A polygonal 2D region of interest (ROI) along the internal surfaces of the cortical shell was drawn and inverted approximately every ten sections for each 3D preloaded image used to generate the microFE models. A dynamic interpolation was applied in between ROIs. The mask was used to identify those points where the DVC and microFE displacement were compared that laid within the cortical shell (the number of points in the cortical shell ranged from 9 to 31 for the different specimens) and those elements with strain beyond yield within the cortical shell.

The largest microFE model contained over 962 million DOF and on average the analysis required approximately 120 minutes to solve in the finite element software Mechanical APDL (*ANSYS*® Academic Research, Release 15.0) using parallel distributed memory (use of a maximum of 64 CPUs and maximum memory of 311Gb).

### Statistics

To remove outliers, the Cook’s distance method was applied to delete any data point with Cook’s distance equal or higher than five times the Cook’s distance mean value for each specimen in each displacement direction [42]. Linear regressions were used to correlate the numerical and experimental values of local displacements and the slope, intercept, and the coefficient of determination (R^2^) were reported. The accuracy of numerical models predictions of local displacements was evaluated through the computation of the root mean square error (RMSE), the RMSE divided by the absolute maximum experimental value (RMSE%), the absolute maximum value of the difference between the predicted and the experimental values (MaxError), and the concordance correlation coefficient (CC [43]).

The absolute percentage difference (%diff_AF) between numerical and experimental values of axial reaction forces was calculated for each specimen for the models solved with a tissue modulus obtained from the literature (E_t_ = 12.0GPa) and a modulus from the back-calculation procedure (E_t_ = 4.6GPa).

## Results

**Fig 2:**
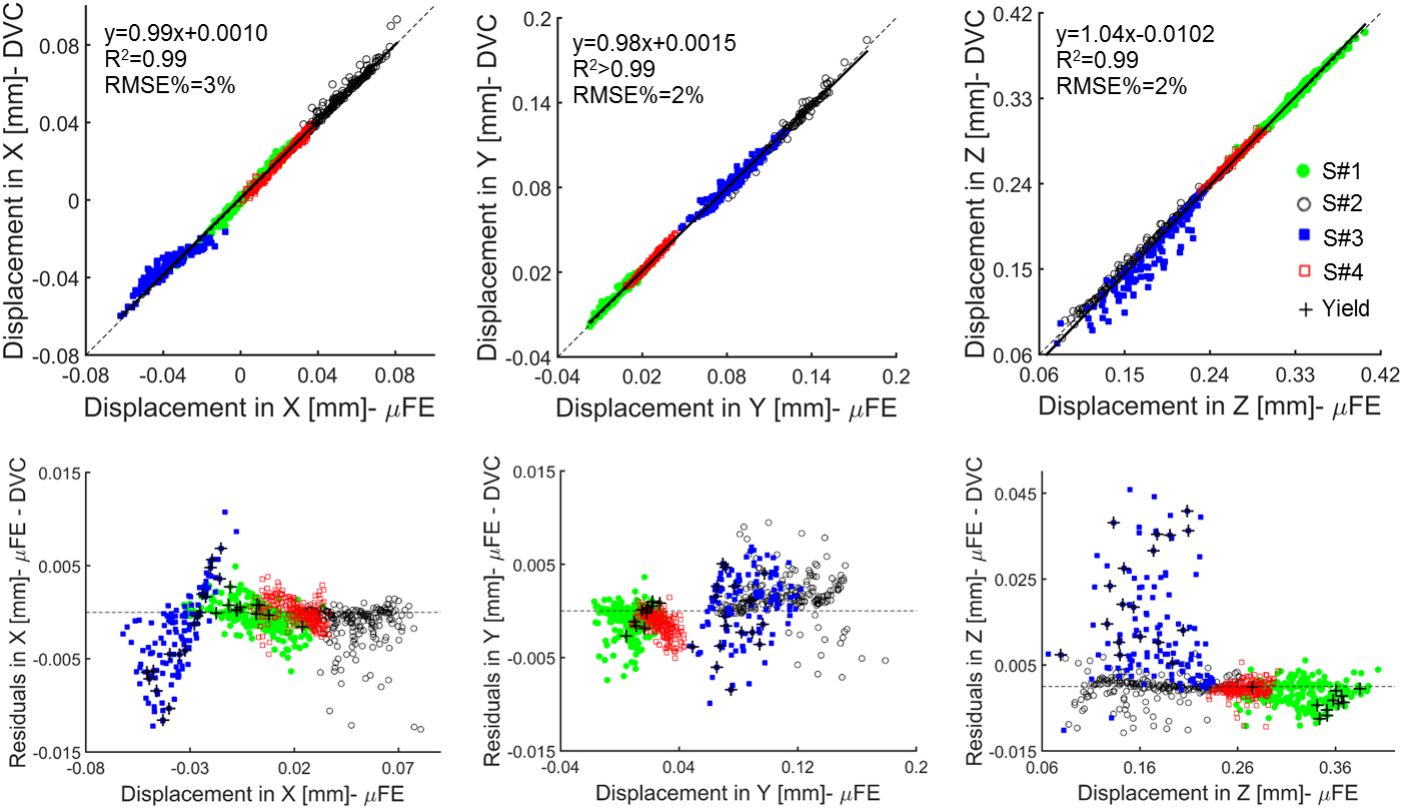
Linear regression and residual analysis estimated between predicted and experimental local displacements for pooled data. Top: correlation between the displacements along the transverse (X, Y) and axial (Z) directions computed by the microFE models and measured experimentally by the DVC approach for the pooled data. Bottom: plots of the residuals. The elements with tensile or compressive strains beyond the yield limits (ɛ_p1Y_ = 7200μɛ and ɛ_p3Y_ = -8000μɛ for vertebral trabecular bone [44]) are reported with black crosses.

MicroFE models predictions of local displacements are reported for models generated with E_t_ = 12.0GPa, but as expected similar results were obtained for E_t_ = 4.6GPa (differences of RMSE% smaller than 0.007% for all the specimens along X, Y and Z directions. S1 Table). From the analysis of local displacements, less than 3.3% of the points were excluded from each specimen by applying the Cook’s distance criterion (Table 3). MicroFE models predictions of local displacements were highly correlated and in agreement with the experimental measurements (R^2^ and CC both ranged between 0.87 and >0.99) (Table 3, Fig 2). In addition, slopes and intercepts of the linear regression analysis were close to the 1:1 relationship for all the directions and for all the specimens (Slope: 0.71 to 1.09, Intercept: -22.10 μm to 4.56μm) (Table 3, Fig 2).

**Table 3:** Linear regression analysis between experimental and predicted local displacements for a tissue modulus E_t_ = 12.0GPa. Data are reported for predictions along the three Cartesian directions (X and Y in a transverse plane, Z in the axial direction) for the individual specimens and for pooled data.

Specimen ID	Direction	Nr. Comparison points (%)	Slope	Intercept [μm]	R^2^	RMSE [μm]	RMSE%	MaxError [μm]	CC^1^
**S#1**	UX	213 (98.6%)	1.05	0.33	0.99	1.35	3.99	6.36	0.99
	UY	215 (99.5%)	0.98	1.12	0.97	1.64	5.25	7.42	0.98
	UZ	215 (99.5%)	0.99	3.25	0.99	2.78	0.70	9.20	0.99
**S#2**	UX	205 (96.7%)	1.02	0.35	0.97	2.31	2.47	12.56	0.98
	UY	209 (98.6%)	1.00	-1.96	0.99	2.31	1.25	9.48	0.99
	UZ	207 (97.6%)	0.99	1.30	>0.99	2.93	1.11	10.79	1.00
**S#3**	UX	130 (99.2%)	0.71	-8.00	0.87	3.11	5.20	12.23	0.87
	UY	130 (99.2%)	0.95	3.85	0.96	3.26	2.72	9.92	0.98
	UZ	131 (100%)	1.05	-22.10	0.91	11.88	5.08	45.86	0.90
**S#4**	UX	226 (98.7%)	1.05	-1.06	0.98	1.25	3.19	4.50	0.99
	UY	226 (98.7%)	1.09	-1.12	0.99	0.97	2.05	5.05	0.98
	UZ	225 (98.3%)	0.99	4.56	0.99	1.69	0.57	9.33	0.99
**Pooled**	UX	774 (98.2%)	0.99	1.04	0.99	2.55	2.74	12.56	1.00
	UY	780 (99.0%)	0.98	1.46	>0.99	2.18	1.18	9.92	1.00
	UZ	778 (98.7%)	1.04	-10.21	0.99	6.96	1.74	45.86	0.99

^1^Concordance Correlation Coefficient according to [43].

For S#1, S#2, and S#4, predictions of local displacements along the axial direction (Z) were more accurate (RMSE% close to 1%) than the predictions computed along the transverse directions (X, Y) (RMSE% in the range 1–5%) (Table 3). For S#3 higher errors were observed along the axial direction, Z, (RMSE% = 3%-5%) and worse correlations were found compared to the other three specimens (0.87<R^2^<0.91 for S#3 and 0.97<R^2^<1.00 for all the others) (Table 3). Maximum differences between numerical and experimental local displacements were lower than or equal to 13μm for S#1, S#2, and S#4 (Table 3). For those specimens the distribution of residuals was homogenous and with an average value close to zero. For S#3 the residuals were more scattered and associated with a systematic overestimation of the predictions of axial local displacements (along Z) up to a maximum of 46μm (Fig 2, Table 3).

**Fig 3:**
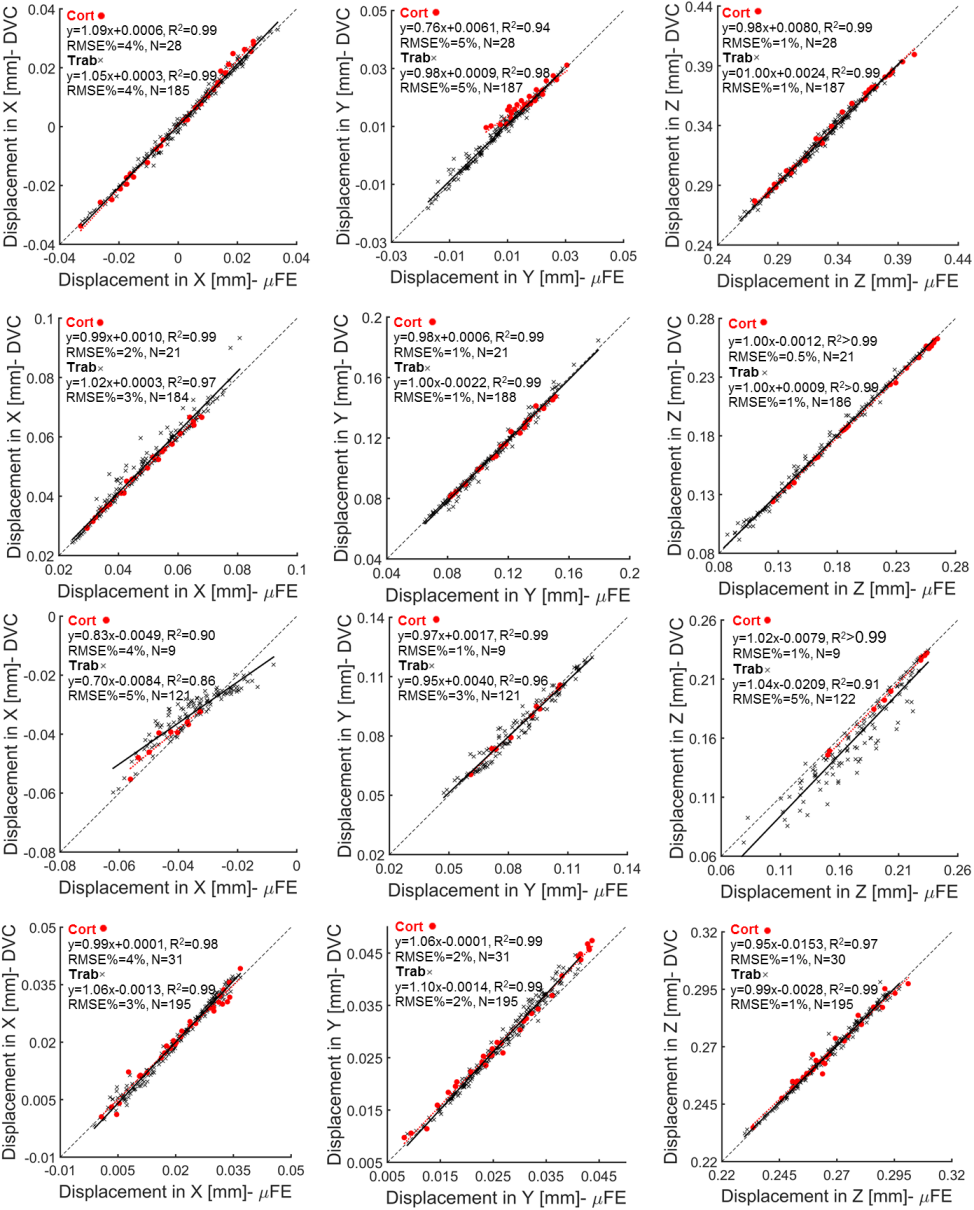
Regression analysis of microFE models predictions of local displacements per specimen and bone type. MicroFE models predictions and DVC measurements computed along the transverse (X, Y) and axial (Z) directions for each specimen within cortical (red circles) and trabecular (black crosses) bone regions.

Similar trends were found for microFE predictions of local displacements in the cortical and trabecular bone regions (i.e. RMSE% between 1% and 5% in the cortical and trabecular bone along transverse directions and RMSE% approximately of 1% for points in the cortical and trabecular regions along the axial direction for all specimens but S#3) (Fig 3 and Table 3). Considering all directions and all specimens, similar correlations were found for microFE predictions performed in the cortical region (0.90≤R^2^<1.00, 0.83≤Slopes≤1.09, and -7.89μm ≤Intercepts≤15.26μm) compared to those obtained in the trabecular region (0.86≤R^2^<1.00, 0.70≤Slopes≤1.10 and -20.92μm ≤Intercepts≤3.96μm) (Fig 3 and S2 Table). In particular, the largest difference between predictions of the cortical and trabecular regions was observed for the axial displacement in S#3 (R^2^>0.99 and RMSE% = 1%, compared to R^2^ = 0.91 and RMSE% = 5% for the trabecular region).

**Fig 4:**
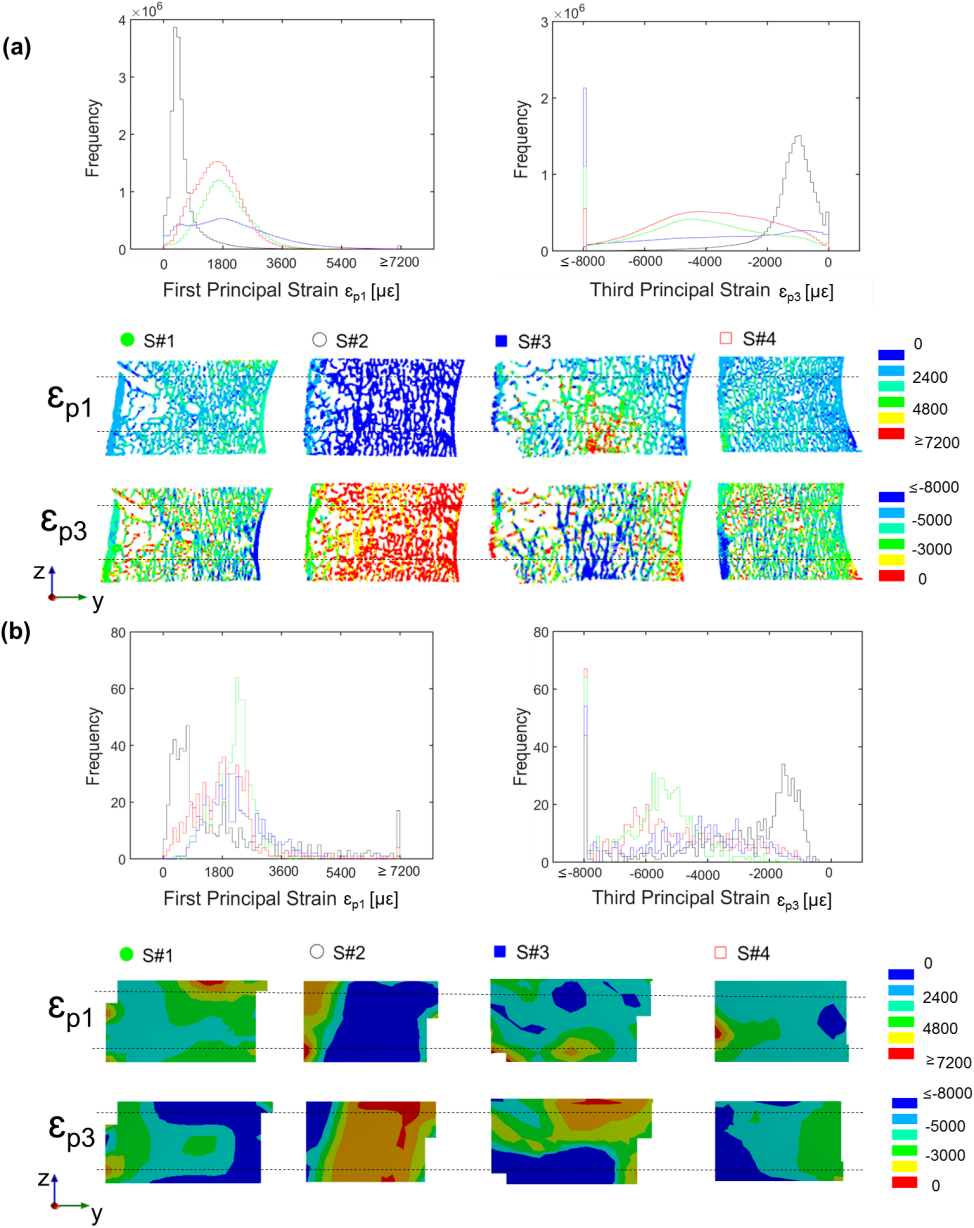
**Distribution of first and third principal strains from microFE models (a) and DVC measurements (b) for each specimen.** For both sub-graphs in the top the frequency plots of the first (tension, ɛ_*p1*_) and third (compression, ɛ_*p3*_) principal strains are reported for the middle portion of each microFE model (a) and for the corresponding region from the DVC analysis (b). The highest and lowest bins represent the number of elements beyond the yield. For both sub-graphs in the bottom the rendering of strain distribution calculated from the microFE models (a) and DVC analysis (b) are reported for a sagittal mid-section (posterior on the left, anterior on the right) for each specimen. Black dashed lines represent the portion of the microFE models and DVC analysis included in the calculation of the frequency plots.

The distribution of the microFE predicted principal strains revealed a predominance of compressive strains for all the specimens. The number of nodes with third principal strain (ɛ_p3_) exceeding the yield value in compression (ɛ_p3Y_) was always larger (range: 0.3%-13% for ɛ_p3Y_ = -8000μɛ) than the number of nodes with first principal strain (ɛ_p1_) exceeding the yield value in tension (ɛ_p1Y_; range: 0.01%-0.3% for ɛ_p1Y_ = 7200μɛ) (Fig 4). S#3 showed the highest percentage of nodes with strain exceeding the compressive yield limit (13%) followed by S#1 (5%), S#4 (2%) and S#2 (0.3%) (Fig 4). In S#3 the high strains were located at the bottom portion of the microFE model, which correspond to the region closer to the experimental platen where the load was applied (Fig 4). In spite of the difference between the dimensions of the cells used for computing the strain with the DVC (cell size approximately 1872μm) and microFE analysis (element size approximately 39μm), similar principal strain distributions were observed between both methods for all the specimens (Fig 4).

**Fig 5:**
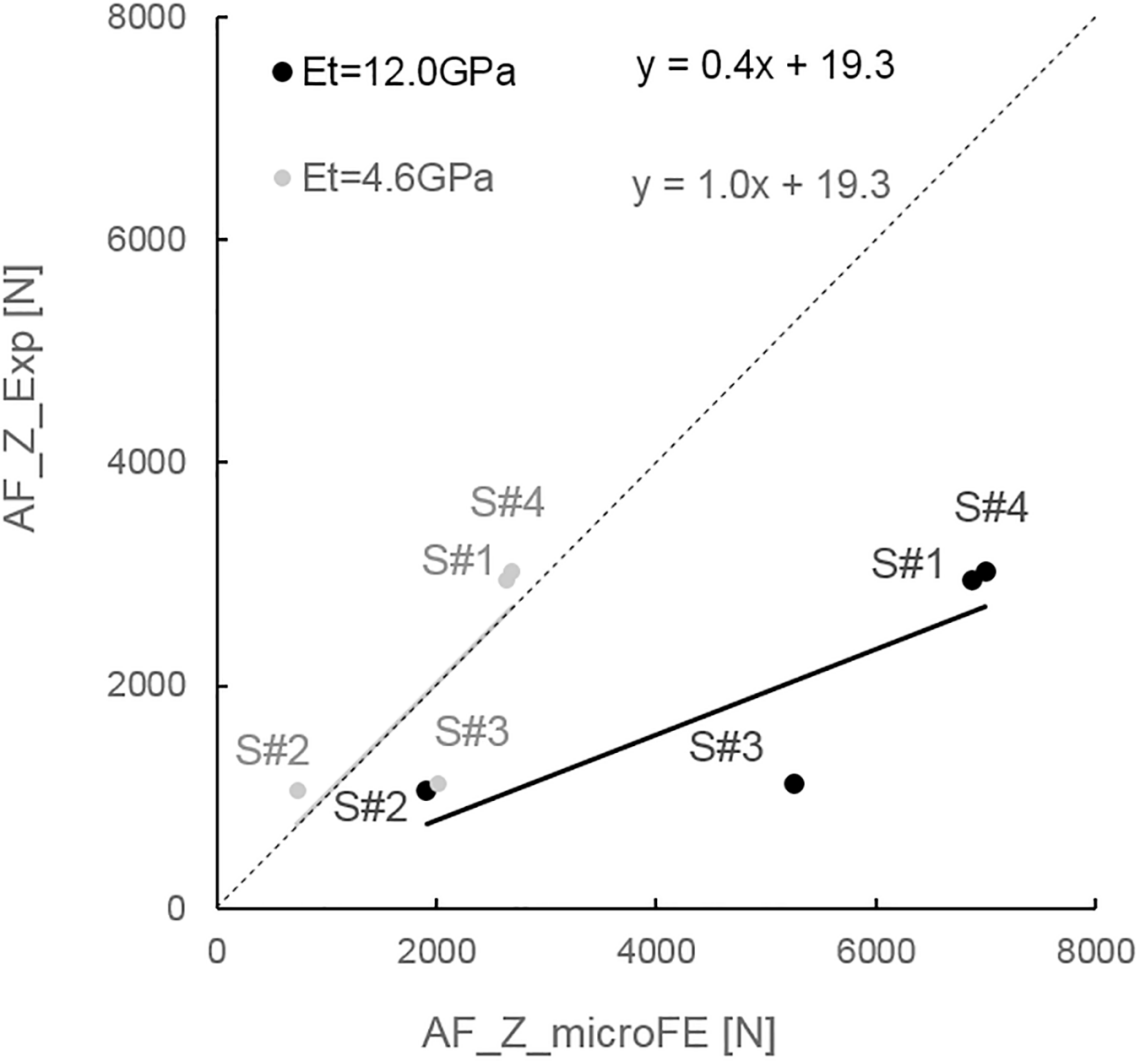
Relationship between numerical (AF_Z_microFE) and experimental (AF_Z_Exp) measurements of axial force for each specimen. Predictive results obtained from models generated with a tissue modulus (Et) equal to 12.0GPa (black) or 4.6GPa (grey).

A higher percentage of cortical elements were found to be deformed beyond compressive yield in S#1 and S#4 (proportion of cortical elements with respect to the total number of elements beyond yield in compression: 2.70% for S#1, 0.00% for S#2, 0.04% for S#3, and 0.55% for S#4). No or a very low number of elements were strained above yield in tension in the cortical shell (proportion of cortical elements with respect to the total number of elements beyond yield in tension: 0.00% for S#1, S#2, and S#3, and 0.01% for S#4). To achieve a good agreement between predicted and measured axial forces the tissue modulus had to be decreased from 12.0GPa to 4.6GPa through a back-calculation procedure (Fig 5).

The axial forces predicted by microFE models with an elastic tissue modulus of 12.0GPa largely overestimated the experimental values (%diff_AF between 80% and 369%, Table 4). For simulations using the back-calculated tissue modulus of 4.6GPa, the percentage differences were smaller, between 10% and 80% (Table 4). For both E_t_ = 12.0GPa and E_t_ = 4.6GPa, S#3 showed the larger residuals.

**Table 4:** Values of axial forces predicted by the microFE models for E_t_ = 12.0GPa and E_t_ = 4.6GPa and experimentally measured, for all specimens. The absolute percentage differences (%diff_AF) between numerical and experimental values are reported.

Specimen ID	AF_Exp [N]	E_t_ = 12.0GPa		E_t_ = 4.6GPa	
		AF_microFE [N]	%diff_AF	AF_microFE [N]	%diff_AF
**S#1**	2953	6881	133%	2643	10%
**S#2**	1060	1910	80%	734	31%
**S#3**	1122	5256	369%	2019	80%
**S#4**	3028	6999	131%	2689	11%

The results are shared in figshare at the following link: https://doi.org/10.15131/shef.data.5121871. The interested reader is encouraged to contact the corresponding author for accessing all the input data and images used in this study.

## Discussion

The aim of this study was to validate microFE models predictions of local displacements against an accurate experimental dataset collected from step-wise *in situ* tests performed on four porcine vertebral bodies. For the first time this analyses was also performed in the trabecular and cortical compartments, separately. Furthermore, due to the uncertainty about the elastic tissue modulus to use in the microFE models based on microCT images with resolution of approximately 40μm, analyses between predicted and measured axial forces for two different tissue moduli were performed.

The results showed that microFE models could predict more than 87% of the variation of local displacements in vertebral bodies in any of the three Cartesian directions (Fig 2), in line with previous investigations performed on trabecular bone specimens by Chen et al. (2017). The predictive error of the microFE models was lower than 13μm (1/3 of the voxel size) for three out of four specimens (Table 3, Fig 3). Smaller errors were observed along the axial direction, which are probably driven by the larger experimental displacements along the direction of compression, Z (RMSE% ranged from 3–5% for UX, and 1–5% for UY and UZ). For three specimens most of the residuals computed for the local displacements were homogeneously distributed and fell within the range of the experimental precision error of the DVC approach (i.e. 3.7μm, as previously reported by Palanca et al. (2016) using similar specimens) (Fig 3). However, for one specimen (S#3) larger differences were found, especially along the axial direction. For that specimen the axial displacements were systematically overestimated by up to 46μm. This overestimation was probably due to the fact that part of S#3 (close to the boundaries of the model, Fig 4) was in the plastic regime (over 13% of the elements were compressed beyond the yield strain of -8000μɛ [44]). The linear microFE modelling approach used in this study could not describe the local plastic behavior of the yielded region. This affected the displacement response also of the surrounding tissues. This specimen may have been compressed above the yield due to its low total bone volume fraction (32.7% vs 40.3–48.6% for the other specimens) and mean trabecular thickness (198μm vs 217–241μm for the other specimens) (Table 2). Further analysis were performed in order to investigate differences between microFE model predictions for cortical and trabecular bone separately. It was observed that microFE models prediction of local displacements performed equally well for both cortical and trabecular bone (RMSE% for cortical and trabecular bone varied from 1% to 5% for transverse directions and were approximately 1% in the axial direction for all specimens but S#3). The absolute maximum errors of microFE models predictions of local displacements ranged between 3μm to 7μm in cortical regions (i.e. 18% the voxel size) while in the trabecular bone it was between 4μm and 46μm with S#3, the specimen which seems to be strained beyond the yield, showing the highest errors (see S2 Table). In fact, in S#3 most of the yielded elements are in the trabecular regions, which is in agreement with the strain distribution observed along the sagittal cross-section of the specimen’s model reported by the DVC (Fig 4). While for three out of four specimens most of the elements strained beyond compressive yield were localized in the trabecular region (range: 70% to 100%), for S#1 the yielded elements were evenly distributed in cortical and trabecular regions (48% in trabecular bone, 52% in the cortical shell), highlighting the variability in strain distributions for the different specimens.

This validation study has focused on the comparison of predicted and measured local displacement, due to the fact that reasonable precision of the DVC approach for strain measurements can be obtained only if large nodal spacing (approximately 50 times higher than the element size of the microFE elements) is used, limiting the spatial resolution of the experimental strain measurement. Nevertheless, a qualitative agreement between the strain distributions measured with DVC and predicted by the microFE models is found for all the specimens (Fig 4). However, direct quantitative comparison between predicted and DVC measured local strains could be only performed by increasing the resolution of the original input images (for example with Synchrotron radiation microCT images [45]).

A reasonable quantitative agreement between the total axial forces predicted by the microFE models and that measured experimentally was achieved only when a back-calculated elastic tissue modulus of 4.6GPa was assigned. This value is much lower than that experimentally measured by wet microindentation tests on adult human bone (mean values around 12.0GPa, Table 1) and lower than that back-calculated in other studies performed on adult human vertebrae (mean values between 5.7GPa and 8.8GPa, Table 1). It is known that the back-calculation compensates not only for actual material properties, but also for potential limitations in the scanning and modeling approaches: partial volume errors, segmentation errors, the use of a Cartesian mesh, and the assumptions of homogenous, isotropic and linear elastic material properties. The quality of the microCT images used for the reconstruction of bone geometry and microstructures is an important factor for the reliability of microFE models. In previous studies the predictions of microFE models of trabecular bone biopsies were found to be sensitive to the segmentation procedure [11,46] and a small changes in the global threshold (e.g. 6% change to the considered optimum value) were associated to large differences (approximately 50% changes) in predictions of global stiffness, with larger effects for specimens with low bone volume fraction. In this study we have investigated the sensitivity of the microFE models in function of the applied global threshold value for predictions of axial forces. Differences of 3% in the threshold value lead to differences in the predicted axial force between 9% and 29% for microFE run with a back-calculated tissue modulus (i.e. 1% <%diff_AF< 20% excluding S#3 for a decrease of 3% in the threshold value; S1 Supporting Information). Contrary to what has been reported in similar studies [11,46], a worse prediction of axial forces by microFE models generated from higher bone volume fraction specimen was observed (i.e. Tot.BV/TV of S#4 equal to 48% and between 33% and 42% for the other three specimens; S1 Supporting Information). This difference can be due to differences in scanning resolution (15μm and 22μm voxel size in those studies) and bone microarchitecture.

From another recent study it was shown that the discretization of bone structures through a tetrahedral mesh provides similar predictions of local displacements, but apparently better local strain estimations compared to standard Cartesian meshes when applied to trabecular bone [47], and may therefore improve the predictions of structural forces. The assumption of local tissue homogeneity seems to have a minor effect on the predictions of microFE models as shown for trabecular bone specimens scanned at a voxel size of 10μm [8] or for vertebral bodies scanned with HR-pQCT with 82μm voxel size [9]. However, it is not clear yet if for microCT scans with approximately 40μm voxel size this approach would be beneficial. Post-yield [10,14,19,48,49], damage [50,51], and viscoelastic [52,53] behaviours have been modelled for trabecular bone specimens, but nonlinear microFE models of whole bones have been limited due to its high computational demand [54,55]. Interestingly, Manda et al. (2016) [56] by using creep-recovery experiments showed that even at lower stress levels trabecular bone experiences both recoverable and irrecoverable local deformations. Such deformations had a faster trend in specimens with a low bone volume fraction, thus underlining the impact of inter-specimen heterogeneity. The specificity of the back-calculated modulus to a set of specimens, images, and models makes the comparison among similar studies difficult. The differences with respect to the study performed by [9] (E_t_ = 8.78GPa) may be due to the different age and species (young porcine vs adult human) and the different resolution of the images used (82μm voxel size in that study vs 39μm voxel size in this study). For a lower scanning resolution (23μm voxel size) Ladd et al. found a back-calculated tissue modulus for trabecular bone samples of human vertebra higher than that found in this study (6.6±1.1GPa, range: 5.4–7.7GPa, N = 5) [15]. However, with similar image resolution (50μm voxel size) Hou et al. found a tissue modulus for human vertebral trabecular bone samples closer to that determined in this study (5.7±1.6GPa; range: 2.7–9.1GPa, N = 28) [16].

The main limitation of this study is the low sample size and the animal origin of the specimens. It remains to be investigated if the different microarchitecture of the human vertebral bodies (i.e. thinner cortical shell and lower density) would affect the predictive ability of microFE models. This detailed validation study limits its applicability to a large sample size and the results obtained from the four specimens confirms the feasibility of this approach. Regarding the effect of using young porcine tissue the assessment is more complicated. In fact, while it is more ethical to perform validations studies on animal tissues, the lack of experimental data reporting the tissue modulus of vertebral bone tissue from young (nine months old) porcine may be an issue. However, the local elastic modulus measured with depth-sensing microindentation in wet conditions from the mid-diaphysis of femurs collected from young pigs at 6–12 months of age (range for osteonal bone: 13.8–19.4GPa; range for interstitial bone: 17.5–20.0GPa; computed from the graphs reported by [57]) and from adult human subjects (mean for osteonal bone: 16.2GPa; mean for interstitial bone: 18.0GPa; computed from the tables reported by [58]) are similar. Therefore, in this study the average elastic tissue modulus reported by [13], who performed measurements on human vertebral tissue is used, assuming small differences between young porcine and adult human local elastic properties. A further limitation is the use of simple (but efficient) microFE models (i.e. Cartesian, homogeneous, linear elastic, and isotropic). Nevertheless, the goal of this study was not to optimize the modelling approach but to show the predictive ability of local displacements and of axial forces for the simplest and most commonly used microFE modelling approach.

In conclusion, the results of this study show that homogeneous linear elastic microFE models can be used to accurately predict the local displacements within both cortical and trabecular bone tissue of vertebral bodies, but at the structural level reasonable predictions of axial forces can be achieved only with properly tuned tissue modulus. The good predictions of local mechanical properties found in this validation study provides a fundamental insight for developing reliable models that link local bone deformation with mechano-regulated cell activity, essential for predicting bone remodeling over time.

## Supporting information

S1 Table Statistical analysis for the linear regressions between experimentally measured displacements and those predicted by microFE models generated with the back-calculated elastic tissue modulus E_t_ = 4.6GPa. Data is reported for predictions along the three Cartesian directions (X and Y in a transverse plane, Z in the axial direction) for all the specimens separately and for pooled data.
(PDF)

S2 Table Additional linear regression analysis between experimental and predicted local displacements for a tissue modulus E_t_ = 12.0GPa performed for the different bone types (i.e. cortical, Cort, and trabecular, Trab, bones). Data are reported for predictions along the three Cartesian directions (X and Y in a transverse plane, Z in the axial direction) for the individual specimens.
(PDF)

S1 Supporting Information (PDF)
