# Supplementary material for: Micro Finite Element models of the vertebral body: Validation of local displacement predictions
Source: PLoS One. 2017 Jul 11;12(7):e0180151. doi: 10.1371/journal.pone.0180151 (PMC5507408; doi:10.1371/journal.pone.0180151)
Supplement: S1 Table — Data is reported for predictions along the three Cartesian directions (X and Y in a transverse plane, Z in the axial direction) for all the specimens separately and for pooled data. (PDF) [file pone.0180151.s001.pdf]

S1 Table. Statistical analysis for the linear regressions between experimentally measured displacements and those predicted by microFE models generated with the back-calculated elastic tissue modulus E_t=4.6GPa. Data is reported for predictions along the three Cartesian directions (X and Y in a transverse plane, Z in the axial direction) for all the specimens separately and for pooled data.

**Specimen ID**	**Direction**	**Nr. Comparison points (%)**	**Slope**	**Intercept [μm]**	**R^2**	**RMSE [μm]**	**RMSE%**	**MaxError [μm]**	**CC[1]**
**S#1**	UX	213 (98.6%)	1.05	0.33	0.99	1.35	3.99	6.36	0.99
	UY	215 (99.5%)	0.98	1.12	0.97	1.64	5.25	7.42	0.98
	UZ	215 (99.5%)	0.99	3.25	0.99	2.78	0.70	9.20	0.99
**S#2**	UX	205 (96.7%)	1.01	0.54	0.97	2.31	2.47	12.28	0.98
	UY	209 (98.6%)	1.00	-1.91	0.99	2.33	1.26	10.05	0.99
	UZ	207 (97.6%)	1.00	0.57	1.00	2.91	1.11	10.49	1.00
**S#3**	UX	130 (99.2%)	0.71	-8.00	0.87	3.11	5.20	12.23	0.87
	UY	130 (99.2%)	0.95	3.85	0.96	3.26	2.72	9.92	0.98
	UZ	131 (100%)	1.05	-22.10	0.91	11.88	5.08	45.86	0.90
**S#4**	UX	226 (98.7%)	1.05	-1.06	0.98	1.25	3.19	4.50	0.99
	UY	226 (98.7%)	1.09	-1.12	0.99	0.97	2.05	5.05	0.98
	UZ	225 (98.3%)	0.99	4.56	0.99	1.69	0.57	9.33	0.99
**Pooled**	UX	774 (98.2%)	0.99	1.03	0.99	2.54	2.72	12.28	1.00
	UY	780 (99.0%)	0.98	1.47	1.00	2.18	1.18	10.05	1.00
	UZ	778 (98.7%)	1.04	-10.75	0.99	6.89	1.72	45.86	0.99

[1]Concordance Correlation Coefficient according to Lin (1989).
