# Supplementary material for: Micro Finite Element models of the vertebral body: Validation of local displacement predictions
Source: PLoS One. 2017 Jul 11;12(7):e0180151. doi: 10.1371/journal.pone.0180151 (PMC5507408; doi:10.1371/journal.pone.0180151)
Supplement: S2 Table — Data are reported for predictions along the three Cartesian directions (X and Y in a transverse plane, Z in the axial direction) for the individual specimens. (PDF) [file pone.0180151.s002.pdf]

S2 Table. Additional linear regression analysis between experimental and predicted local displacements for a tissue modulus E_t=12.0GPa performed for the different bone types (i.e. cortical, Cort, and trabecular, Trab, bones). Data are reported for predictions along the three Cartesian directions (X and Y in a transverse plane, Z in the axial direction) for the individual specimens.

Specimen ID	Direction	Nr. Comparison points (%)	Bone sites	Nr. Comparion points per bone site	Slope	Intercept [μm]	R^2	RMSE %	MaxError [μm]
**S#1**	UX	213 (98.6%)	Cort	28	1.09	0.63	0.99	4.27	6.36
			Trab	185	1.05	0.31	0.99	3.93	4.93
	UY	215 (99.5%)	Cort	28	0.76	6.10	0.91	4.86	6.98
			Trab	187	0.98	0.94	0.98	4.83	7.42
	UZ	215 (99.5%)	Cort	28	0.98	7.96	0.99	0.69	7.47
			Trab	187	1.00	2.38	0.99	0.70	9.20
**S#2**	UX	205 (96.7%)	Cort	21	0.99	0.99	0.99	1.52	2.67
			Trab	184	1.02	0.32	0.97	2.55	12.56
	UY	209 (98.6%)	Cort	21	0.98	0.57	0.99	1.25	4.37
			Trab	188	1.01	-2.20	0.99	1.28	9.48
	UZ	207 (97.6%)	Cort	21	1.00	-1.17	1.00	0.47	4.36
			Trab	186	1.00	0.89	1.00	1.15	10.79
**S#3**	UX	130 (99.2%)	Cort	9	0.83	-4.94	0.90	3.64	7.03
			Trab	121	0.70	-8.39	0.86	5.18	12.23
	UY	130 (99.2%)	Cort	9	0.97	1.67	0.99	1.10	2.11
			Trab	121	0.95	3.96	0.96	2.81	9.92
	UZ	131 (100%)	Cort	9	1.02	-7.89	1.00	0.56	5.76
			Trab	122	1.04	-20.92	0.91	5.16	45.86
**S#4**	UX	226 (98.7%)	Cort	31	0.99	0.12	0.98	4.02	4.50
			Trab	195	1.06	-1.28	0.99	3.09	3.79
	UY	226 (98.7%)	Cort	31	1.06	-0.09	0.99	2.05	3.91
			Trab	195	1.10	-1.40	0.99	2.14	5.05
	UZ	225 (98.3%)	Cort	30	0.95	15.26	0.97	0.83	7.29
			Trab	195	0.99	2.85	0.99	0.51	9.33
