# Supplementary material for: Micro Finite Element models of the vertebral body: Validation of local displacement predictions
Source: PLoS One. 2017 Jul 11;12(7):e0180151. doi: 10.1371/journal.pone.0180151 (PMC5507408; doi:10.1371/journal.pone.0180151)
Supplement: S1 Supporting Information — (PDF) [file pone.0180151.s003.pdf]

S1 Supporting Information

In order to analyze the sensitivity of the outputs of the microFE models on the chosen global threshold value a further investigation was performed for models generated with the back-calculated elastic tissue modulus (E_t= 4.6GPa).

Variations of ±3% from the optimal threshold values of each specimen were considered as the maximum range that would still allow reasonable reconstructions of bone tissue microstructures for all specimens, based on visual inspection of binary and original gray scale images.

For each specimen three models were generated: one with the optimal threshold value, one with the optimal threshold value increased of 3% and one with the optimal threshold value decreased of 3%. Predictions of local displacements and axial forces against experimental values were performed as described in the manuscript. The accuracy of predicted and experimental local displacements were compared along X, Y, and Z (UX, UY, and UZ) by reporting the Root Mean Square Error percentage (RMSE%). Whereas predicted axial reaction forces were compared to the experimental values (percentage difference, %diff_AF).

Table. Effect of a 3% variation (±3%Th_opt) in the optimal threshold values (Th_opt) of each specimen on microFE models predictions of local (RMSE%_Ui range for UX, UY, and UZ) and structural (%diff_AF) properties using E_t=4.6GPa.

Specimen ID	%diff_AF			RMSE%_Ui		
	-3%Th_opt	Th_opt	+3%Th_opt	-3%Th_opt	Th_opt	+3%Th_opt
**S#1**	1%	10%	19%	1%-5%	1%-5%	1%-5%
**S#2**	1%	31%	52%	1%-2%	1%-2%	1%-2%
**S#3**	95%	80%	67%	3%-5%	3%-5%	3%-5%
**S#4**	20%	11%	36%	1%-3%	1%-3%	1%-4%

A variation of 3% in the threshold value did not affected the predictive power of microFE models predictions of local displacement (i.e. largest difference in RMSE% equal to 0.38%). On the other hand, the microFE models predictions of axial force were very sensitive to small changes in the threshold value. Relative changes

of 3% in the threshold lead to differences in microFE models predictions of axial forces ranging from 9% to 29%.
